# A Hitchhiker’s Guide to Myeloid Cell Subsets: Practical Implementation of a Novel Mononuclear Phagocyte Classification System

**DOI:** 10.3389/fimmu.2015.00406

**Published:** 2015-08-11

**Authors:** Martin Guilliams, Lianne van de Laar

**Affiliations:** ^1^Laboratory of Immunoregulation, VIB Inflammation Research Center, Ghent University, Ghent, Belgium; ^2^Department of Respiratory Medicine, University Hospital Ghent, Ghent, Belgium

**Keywords:** nomenclature, dendritic cells, macrophages, monocytes, classification

## Abstract

The classification of mononuclear phagocytes as either dendritic cells or macrophages has been mainly based on morphology, the expression of surface markers, and assumed functional specialization. We have recently proposed a novel classification system of mononuclear phagocytes based on their ontogeny. Here, we discuss the practical application of such a classification system through a number of prototypical examples we have encountered while hitchhiking from one subset to another, across species and between steady-state and inflammatory settings. Finally, we discuss the advantages and drawbacks of such a classification system and propose a number of improvements to move from theoretical concepts to concrete guidelines.

## Introduction

In the science fiction series created by Douglas Adams (1), the Hitchhiker’s Guide to the Galaxy starts as follows: “*Space is big. Really big. You just won’t believe how vastly hugely mind-boggling big it is. You may think it’s a long way down the road to the chemist’s, but that’s just peanuts to space*.” Given the complexity of the mononuclear phagocyte (Star)system (MPS), one could easily give a similar warning to readers who are trying to make some sense of the huge number of hypothetically distinct dendritic cell (DC) and macrophage (MΦ) subsets. At the last International DC Symposium (DC2014, Tours – France), we counted at least 28 different DC subsets that were described using various surface markers and nomenclature systems in distinct species. If one would add the different MΦ subsets and the Cytof technology allowing to measure the expression of more than 30 different surface markers per cell, one could with a bit of luck end up with “42” as answer to the ultimate myeloid question of how many mononuclear phagocyte subsets exist in life, the universe, and everything. Although this would be great for fans of Douglas Adams, without Babel Fish to help us make some sense of so many different subsets, this evolution will not be beneficial for communication among myeloid cell experts, let alone for the communication toward pharmaceutical companies, scientific editors, medical doctors, or graduate students. We will here try to simplify this apparent complexity through a number of practical examples and theoretical concepts. Having hitchhiked from MΦ to DC labs studying myeloid cells in various tissues and in distinct inflammatory conditions, we would, in accordance with the Hitchhiker’s Guide to the Galaxy, advise the following: do not panic and bring your towel along.

## Members of the Mononuclear Phagocyte System

**Figure 1:**
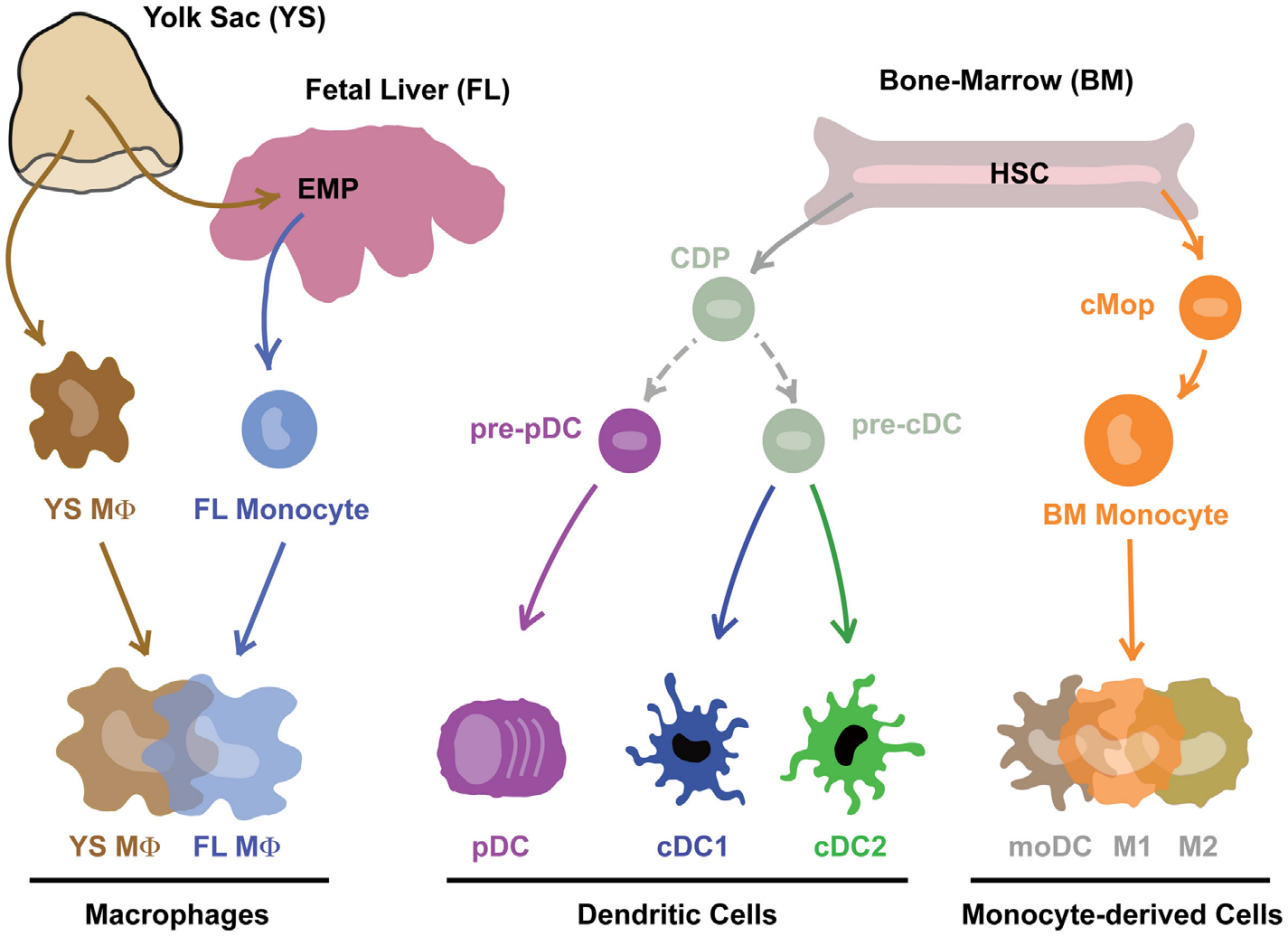
**Mononuclear phagocytes and their precursors**. Note that this is work in progress and technical advances such as single-cell RNASeq and barcoding will in the near future prove or disprove many aspect of this theoretical scheme.

In the original MPS model proposed by Ralph van Furth, James Hirsch, and Zanvil Cohn, MΦs were proposed to derive from circulating monocytes (2). A couple of years later, Ralph Steinman and Zanvil Cohn identified DCs, which were also included in the MPS (3). The fact that DCs could be derived from human and mouse monocytes in GM-CSF-driven *in vitro* cultures (4–8) and *in vivo* upon inflammation or in barrier tissues (9–15) supported this concept. For a historical overview of the MPS field, we redirect the readers to the review of Simon Yona and Siamon Gordon in this issue (16). The identification of mouse hematopoietic precursors committed to the DC lineage called the common DC progenitors (CDPs – giving rise to pDCs and cDCs) and pre-cDCs (giving rise to cDCs) that are distinct from monocytes and can give rise to the so-called conventional DCs (cDCs) induced a first conceptual revolution in the field (12, 17–20). Moreover, Flt3-L, and not GM-CSF, was shown to be critically involved in the development of cDCs *in vitro* (8, 21–23) and *in vivo* (24–28). Recently, two additional committed precursors were identified in mice: the pre-pDC precursor that preferentially differentiates into pDCs (29), and the monocyte-committed common monocyte progenitor (cMop) (30). Importantly, the human equivalent of the pre-cDC, CDP, and cMop was recently identified (31, 32). A second conceptual revolution in the field was driven by the finding that most tissue-resident MΦs do not derive from circulating HSC-derived monocytes but develop from embryonic precursors, i.e., the yolk-sac MΦs (YS MΦs) or fetal liver (FL) monocytes (33–39). The relative contribution of YS MΦ-derived and FL monocyte-derived MΦs seems to vary from one organ to another (40–42). It was recently demonstrated that almost all MΦs have a YS origin [either directly from YS MΦs or through YS-derived EMPs (39)]. This may seem in contradiction with the proposed partial origin from FL monocytes (35, 43). However, it is now clear that YS-derived EMPs seed the FL and go through a FL monocyte intermediate before differentiating into most tissue-resident MΦs (44), reconciling most of the apparent discrepancies in the field. Together, these findings have challenged the MPS dogma and revealed that most DCs and MΦs derive from distinct committed precursors rather than from circulating HSC-derived monocytes (Figure 1).

## Revisiting the Classification of Mononuclear Phagocytes

Historically, mononuclear phagocytes were classified as DCs or MΦs based on a restricted set of surface markers (CD11c and MHCII for DCs versus F4/80 for MΦs), proposed functional specialization (antigen-presentation and migration to lymph nodes for DCs versus phagocytosis for MΦs) and/or morphological features (dendritic-shaped cells for DCs versus large vacuolar cells for MΦs). However, these features are often not mutually exclusive. For example, although CD11c and MHCII are typically associated with DCs, alveolar MΦ are CD11c^hi^ and MHCII is expressed by intestinal MΦs (35, 45). Ideal surface markers allowing identification of the distinct myeloid cell subsets across tissues and species are still incomplete. Markers typically associated with some myeloid cell subsets can be lost or acquired by other subsets. The monocyte-associated marker Ly-6C is rapidly down-regulated on many monocyte-derived cells (MCs) upon entrance in the tissues (45–48) and is expressed on pDCs (and lowly expressed on some cDCs). The pDC-associated marker mPDCA1 (stained with 120G8) can be acquired by MCs during inflammation (49). Alveolar MΦs (50) and Kupffer Cells (unpublished data) can upregulate CD11b during inflammation. Finally, BDCA3 is expressed on both human cDC1s and MCs (51). Thus, the inability to consistently identify myeloid cell subsets irrespective of tissue, species, or inflammatory state makes surface markers unattractive as basis for classification.

We would also propose to avoid a classification based primarily on functional specialization. First, each myeloid cell subset can perform more than one prototypical function. MΦs are often linked to phagocytosis of dead cells and pathogens but also have important immunomodulatory and metabolic functions. Second, subsets can acquire or lose functional capacities during inflammation as recently demonstrated for cDC2s that acquire cross-presentation capacities upon TLR stimulation (52). Therefore, we propose to disregard function as a basis for classifying cells.

Instead of surface markers, functional specialization, or morphology, we have recently suggested to classify cells based on their cellular origin, which could allow a more robust classification system (53). This would yield three big groups of cells (Figure 1): (i) embryonic progenitor-derived MΦs, (ii) CDP-derived DCs (that would be subdivided into cDC1s, cDC2s, and pDCs), and (iii) MCs. As these precursors have now been identified in both the mouse and the human, this allows one classification system across tissues and species.

**Figure 2:**
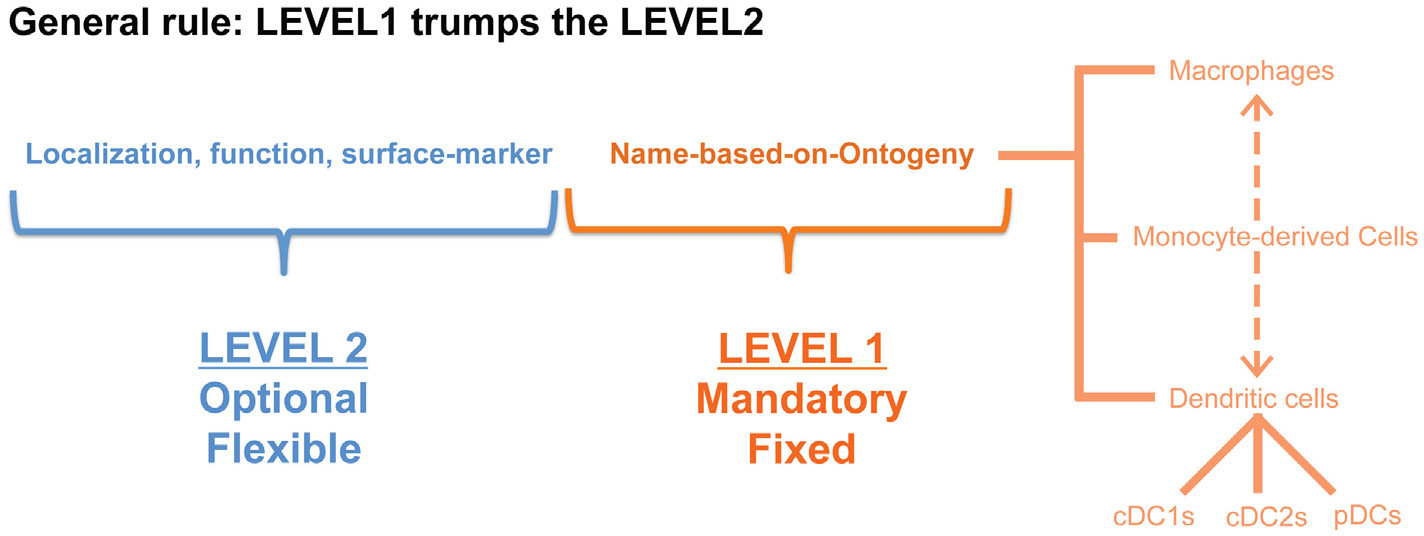
**A nomenclature system in two levels would have the advantage that cells can be first classified based on a restricted set of names (in this proposition according to their cellular origin: MΦ, MC, cDC1, cDC2, pDC) that would be applicable across species and across tissues, but the second level would still allow some flexibility to denote a distinct activation state or localization**.

Although precursor-based classification would provide a robust and species-conserved system, at the end of the day the function of the cells is what really matters for converting our knowledge into therapeutic advances for patients. Regrouping all the DCs into three big subsets of cDC1s, cDC2s, and pDCs will thus have the disadvantage of lumping together cells that may be in very different functional activation states. Similarly, MCs have been shown to be particularly plastic cells (54). Therefore, we propose to add a second classification level to the fixed ontogeny-based Level1 (Figure 2). Addition of a Level2 allows specification of the cellular activation state, the micro-anatomical localization or simply the surface markers utilized to identify the cells in a particular study. Of note, when defining the Level2 it will be important to avoid generalizations as a given function is often performed by only a fraction of the cells studied. We would thus propose to restrict the Level2 to objective criteria that can be measured at the single-cell level.

## Practical Implementation for DCs

**Figure 3:**
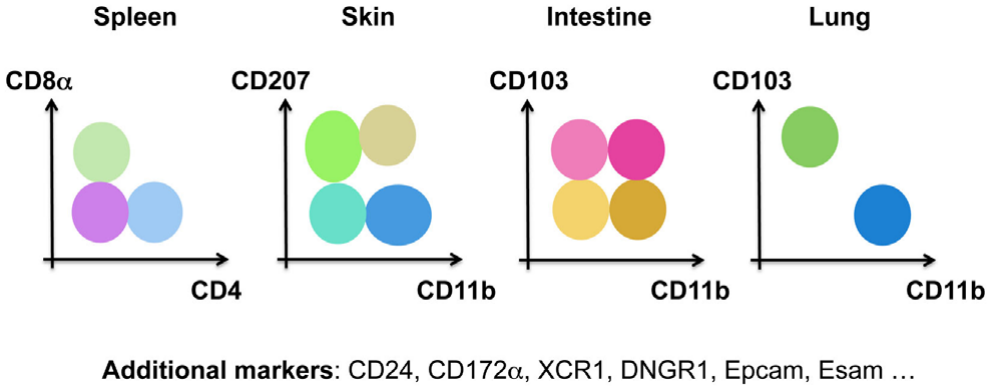
**Murine DCs have been subdivided into many different subsets based on distinct surface markers in the spleen, the skin, the intestine, and the lung**.

Historically, DCs were divided into subsets based on surface markers that differed between tissues and species, such as CD207 (Langerin) in the skin, CD103 (IntegrinαE) in the intestine, CD11b (IntegrinαM) in the lungs, CD4/CD8α in the spleen, and CD24/CD172α for *in vitro* differentiated DCs (Figure 3). Human DCs, on the other hand, have been divided into CD141^+^ (BDCA3) and CD1c^+^ (BDCA1) DCs. pDCs are identified by the expression of BDCA4 and BDCA2 in human, but by B220, mPDCA1 (BST2, recognized by 120G8), or Siglec-H in mice (53). Technical advances in multi-color flow cytometry have made matters worse with evermore “novel DC subsets” based on the expression of additional surface markers. By comparing the gene-expression profile of DCs isolated from various tissues and species, one can appreciate three big clusters of DCs (55–61). The pDC cluster includes mouse PDCA1^+^ pDCs and human BDCA2^+^BDCA4^+^ pDCs. The cDC1 cluster comprises dermal CD207^+^CD103^+^ cDC1s, lung CD103^+^CD11b^−^cDC1s, splenic CD8a^+^CD4^−^ cDC1s, intestinal CD103^+^CD11b^−^ cDC1s and human blood BDCA3^+^ cDC1s. Dermal CD207^−^CD11b^+^ cDC2s, lung CD103^−^CD11b^+^ cDC2s, splenic CD8a^−^CD4^+^ cDC2s, intestinal CD103^+^CD11b^+^ cDC2s and human blood BDCA1^+^ cDC2s form the cDC2 cluster (62, 63). This bio-IT-driven analysis also revealed that within the cDC population, XCR1 and Sirpα are, respectively, expressed by all cDC1s and cDC2s across tissues, allowing an improved identification of these cells (51, 64–70). Note, however, that Sirpα is also expressed by other myeloid cells than cDC2s, showing the need for correct cDC identification prior to using this marker to distinguish cDC2s from cDC1s. Strikingly, this gene-expression-based division is supported by the existence of distinct pre-committed precursors (29, 71, 72) and by differential developmental transcription factor requirement of cDC1s, cDC2s, and pDCs in the mouse. cDC1s, but not cDC2s, require BATF3 (71, 73, 74), ID2 (28, 75, 76), NFIL3 (77), and IRF8 (28, 71, 78–80) for their development, while cDC2s, but not cDC1s, are dependent on RELB (81), RBPJ (82), and IRF4 (79, 83–85). pDC development has been shown to be driven by E2-2 (86, 87).

**Figure 4:**
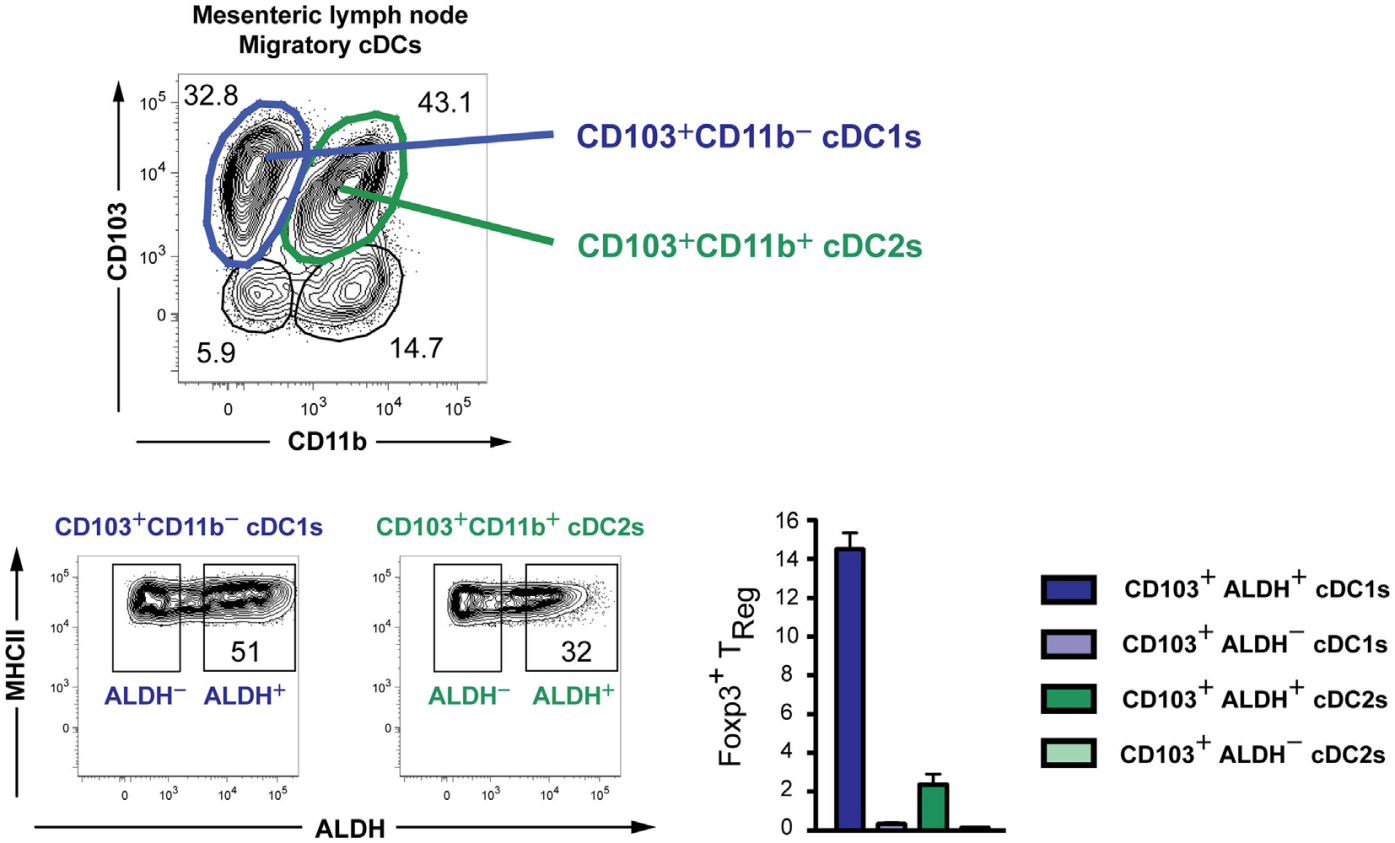
**Existence of subpopulations with distinct retinoic acid-producing capacities within both cDC1s and cDC2s in the mesenteric lymph nodes of mice**. The capacity to produce retinoic acid was measured by the Aldefluor kit (91). DCs were sorted, loaded with the ovalbumin-peptide, and co-cultured *in vitro* with naïve OTII cells to measure the induction of Foxp3 on these cells.

The subdivision of DCs in three distinct Level1 groups is thus supported by their gene-expression profiles, cellular origin, and transcription factor requirement. However, these cells can acquire a distinct functional activation state from one tissue to another and in distinct inflammatory settings, underlining the need for a Level2 system. This can be illustrated by the capacity of intestinal cDCs to produce retinoic acid and promote the generation of induced regulatory T cells (iT_REGs_) (88–91). Identification of DCs with superior iT_REG_ inducing ability is clinically relevant as the prevalence of food allergies, celiac disease and inflammatory bowel diseases is currently rising throughout the western world. Originally, it was described that CD103^+^ but not CD103^−^ intestinal DCs excel in iT_REG_ generation in a retinoic acid-dependent manner (89, 90). It is now clear that CD103^+^ intestinal DCs comprise two ontogenically distinct subsets, CD103^+^CD11b^−^ cDC1s and CD103^+^CD11b^+^ cDC2s (74). Interestingly, rather than being associated with either of the two subsets, about half of the intestinal CD103^+^CD11b^−^ cDC1s were shown to possess the capacity to produce retinoic acid, while only one-third of the cDC2s do (91). Moreover, on a per cell basis retinoic acid producing CD103^+^ cDC1s were the best at inducing iT_REGs_ (Figure 4). These data reveal that CD103^+^ cDC1s, although broadly considered as a homogeneous subset, consist of 50% cells that are very efficient at inducing iT_REGs_ and 50% cells that are not. Interestingly, dermal cDC2s have higher retinoic acid-dependent iT_REG_ induction activity than dermal cDC1s (91). We hypothesize that this functional heterogeneity may be explained by the existence of distinct micro-environments within organs, inducing diverse functional modules on DCs. The finding that important functional modules can be acquired by only a fraction of cDC1s and/or cDC2s, which can moreover differ from one organ to another, illustrates the need for a Level2 nomenclature for DCs.

Another example of functional heterogeneity within DCs concerns the cDC2s. Splenic cDC2s contain a subpopulation that expresses CD4 and is specifically localized in the bridging channels (92). This localization has been shown to be EBI2-driven and essential to drive antibody production by B cells. The development of these CD4^+^ cDC2s is Notch2 dependent. Note also that Notch2 deficiency is associated with defects in T_H_17 induction (93, 94). In addition, it was found that KLF4 controls the development of a subpopulation of CD24^lo^CD11b^lo^Sirpα^hi^ cDC2s in the dermis (95). Importantly, loss of KLF4 was associated with loss of T_H_2 induction. Thus, although cDC2s have been proposed to excel at both the induction of T_H_2 (47, 96) and T_H_17 responses (84, 85, 93), it may well be that these functional modules are in fact expressed by subpopulations of cDC2s (controlled by KLF4 and Notch2, respectively). In conclusion, although the current knowledge of early DC development in the bone-marrow seems to support only three big groups of DCs (cDC1s, cDC2s, and pDCs), it appears that a second layer of tissue-specific signals imprint operative gene modules on a fraction of DCs. Depending on their micro-localization, subpopulations of cDC1s, cDC2s, or pDCs will acquire distinct functional properties, requiring a flexible Level2 to classify and describe functionally distinct subpopulations.

**Figure 5:**
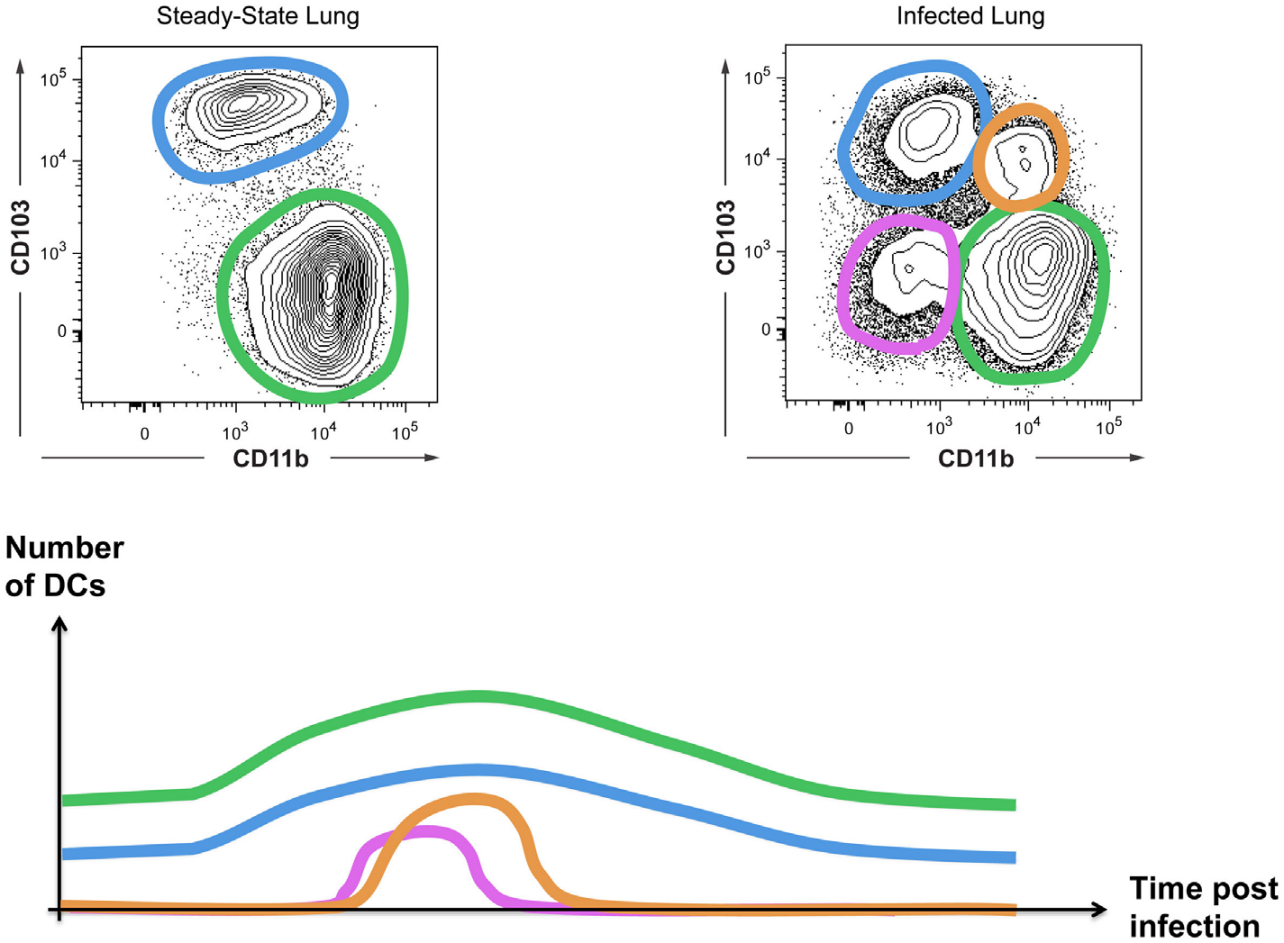
**Inflammation can induce the appearance of “novel” DC subsets**. CD103 and CD11b expression on cDCs from uninfected or influenza-infected lungs are shown. The appearance of CD103^+^CD11b^+^ DCs and CD103^−^CD11b^−^ DCs is transient as schematically represented.

A final example of a need for a Level2 classification involves inflammation-induced changes of surface marker expression. When mice are infected with the influenza virus, there is a transient change in the CD103 versus CD11b expression profile of lung cDCs, yielding four instead of two lung DC subsets (Figure 5). If one considers these as four distinct DC subsets, one could conclude that influenza infection disrupts hematopoiesis in the bone-marrow, as has been shown for Toxoplasma infection (97). Alternatively, these novel CD103/CD11b expression patterns may represent distinct local activation states of cDC1s or cDC2s. We have studied the cellular origin of the “novel” DC subsets arising during influenza infection (Neyt et al., manuscript in preparation). Our preliminary data suggest that CD103^+^CD11b^+^ cells are cDC2s that acquire CD103 expression during inflammation rather than a completely new subset. In conclusion, although we cannot rule out the existence of additional DC subsets that specifically develop during inflammation, when in doubt we propose to first evaluate whether cells with a novel surface receptor expression profile represent a different activation state of cDC1s or cDC2s before assuming the existence of a novel cDC3.

## Practical Implementation for Embryonic Macrophages

In our classification system based on ontogeny all mononuclear phagocytes of embryonic origin are grouped together under a single Level1 as “macrophages” (Figure 1). This includes liver-resident Kupffer cells, brain-resident microglial cells, lung-resident alveolar MΦs but also epidermis-resident Langerhans cells. In effect, this would thus classify Langerhans cells as MΦs and not as DCs, based on the fact that these cells derive from embryonic precursors that seed the epidermis around birth and then self-maintain throughout life (43, 98). We propose to keep the historical names for MΦs with undisputed identities. Mouse Kupffer cells, for example, do not require a different nomenclature since these cells have a well-defined cellular origin [embryonic (34, 38, 39)], localization (i.e., the liver sinusoids), and gene-expression profile (99, 100). However, we would like to emphasize that not any F4/80^+^ cell in the liver should be categorized as Kupffer cell. MCs infiltrating the liver during acetaminophen-induced injury also express F4/80 but are short-lived and acquire a gene-expression profile that is strikingly different from resident Kupffer cells (100). Similarly, MCs infiltrating the central nervous system during inflammation are short-lived and do not acquire the specific gene-expression profile of embryonic microglia (101–103). As such, any MΦ-like cell in the liver or the brain should not be classified as Kupffer cell or microglia, respectively, as is often the case. Unfortunately, tools to correctly distinguish MCs from resident MΦs have long been lacking. In a way, this is surprising given the huge difference in gene-expression profile between resident embryonic MΦs and recruited MCs in these disease models. We have now identified several surface markers that are expressed by Kupffer cells but not MCs recruited during liver injury (Scott et al. manuscript in preparation) and we expect that given the striking heterogeneity of tissue-resident MΦs (104, 105) many of these MΦ-specific markers will be found. This will facilitate the correct classification of these cells and pave the way toward unraveling the functional differences between recruited MCs and tissue-resident embryonic MΦs during inflammation.

## Practical Implementation for Monocyte-Derived Cells

**Figure 6:**
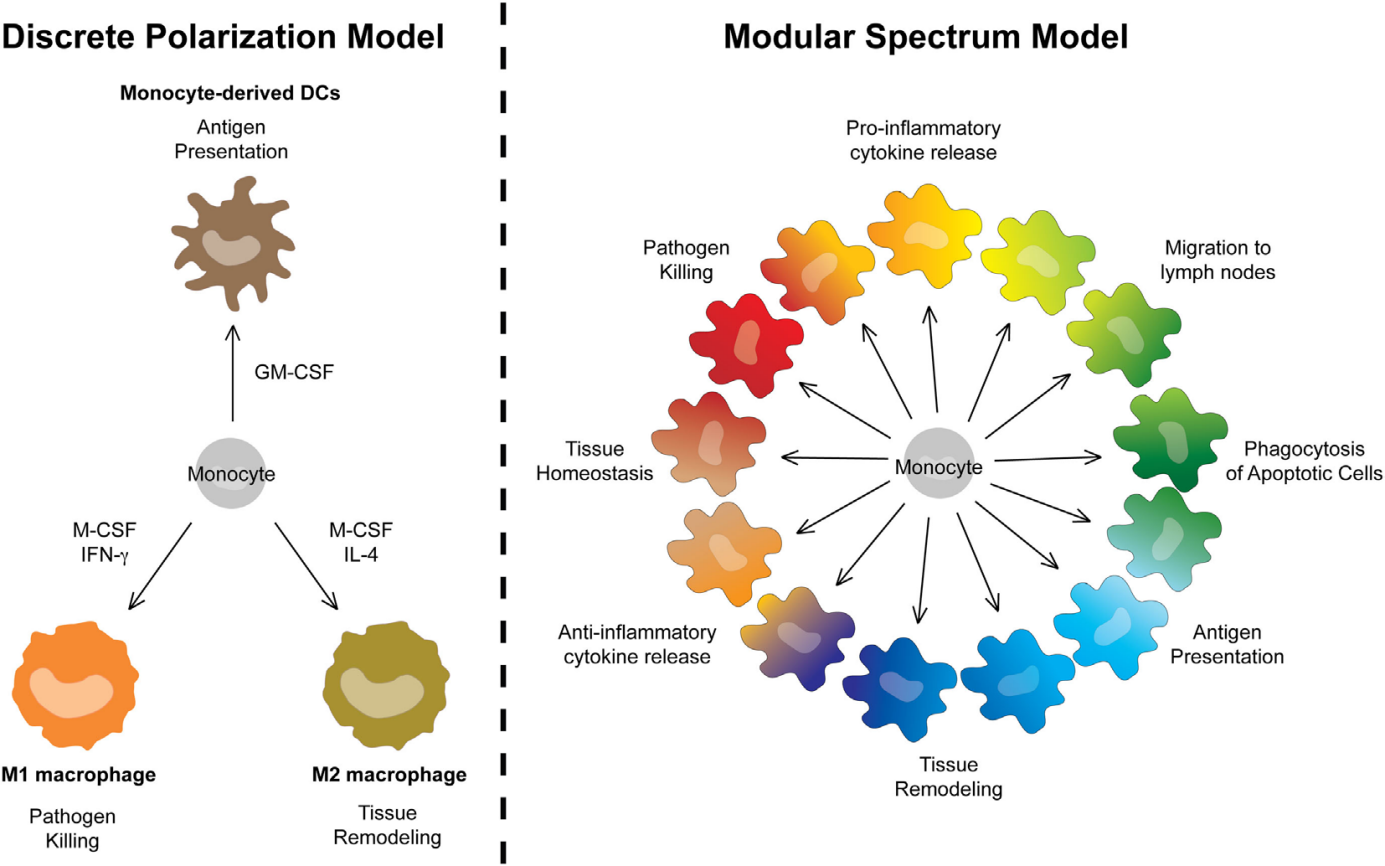
**A modular spectrum model for monocyte-derived cells**. Replacement of the polarized three-way M1/M2/moDC model by a spectrum model in which bacterial killing, wound-healing, and antigen-presentation represent only three of many functional modules that can be acquired by MCs.

Monocytes are particularly plastic cells. This can be appreciated using *in vitro* culture systems. Monocytes cultured with GM-CSF express some DC-like characteristics and have therefore long been referred to as moDCs. By contrast, culturing monocytes with M-CSF induces their differentiation into MΦ-like cells (moMΦs). Adding IL-4 or IFN-γ to M-CSF cultures further polarizes MCs into the so-called “classically activated MΦs/M1s” or “alternatively activated MΦs/M2s” (106), with strikingly different gene-expression profiles and metabolic modules (107). In a nomenclature system based on ontogeny, moDCs, M1s, or M2s are however first classified as MCs (Level1). In theory, this does not prevent further Level2 classification as “dendritic MCs,” “classically activated MCs,” or “alternatively activated MCs.” However, we feel this polarized classification implies functional characteristics that are often not assessed experimentally. For example, MCs classified as M1s are typically associated with pathogen killing, M2s with wound healing, and moDCs with antigen-presentation (Figure 6). However, the identification of “dendritic MCs/moDCs,” “classically activated MCs/M1s,” or “alternatively activated MCs/M2s” *in vivo* turned out to be very challenging. In fact, profiling of MCs isolated from various inflamed tissues or *in vitro* culture systems reveals that monocytes can acquire a much broader transcriptional repertoire than suggested by the three-way M1/M2/moDC model. In recent efforts to further characterize the heterogeneity of MC activation states, Schultze and colleagues compared the gene-expression profile of MCs stimulated with a vast array of cytokines and TLR ligands. Instead of yielding a polarized model, the unbiased bio-informatics-driven clustering approach revealed a spectrum model (54). In our view, this spectrum model can be taken one step further and include the “dendritic MCs/moDCs” derived from GM-CSF-induced bone-marrow cultures as yet another extreme of the continuum of cellular faiths that can be acquired by monocytes. Rather than unique end points, bacterial killing, wound-healing, and antigen-presentation represent three of many functional modules that can be acquired by MCs in a spectrum model that can be graphically represented by a continuous circle (Figure 6).

**Figure 7:**
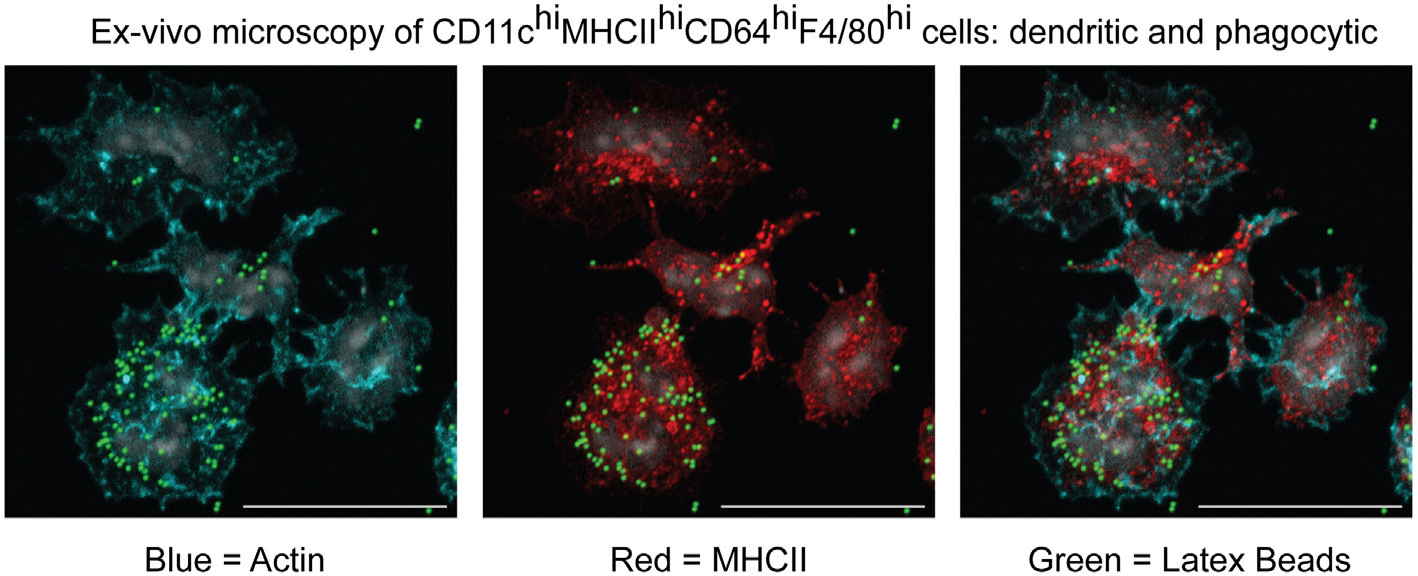
**Microscopic characterization of MCs isolated from inflamed mesenteric lymph nodes in an experimental model for colitis**. CD11c^hi^MHCII^hi^CD64^hi^F4/80^hi^ cells were sorted and cultured *in vitro* to evaluate their morphology and capacity to phagocytose latex beads (45).

One important consequence of the herein-described classification would be the regrouping of moDCs and moMΦs under a single MC Level1. We feel this will represent an improvement for the field due to the lack of clear, mutually exclusive features that can be used to objectively separate moDCs from moMΦs (53). This problem can be illustrated by the MCs present within inflamed mesenteric lymph nodes during an experimental model for colitis (45). In this study, we called these cells moMΦs because they were CD64^hi^F4/80^hi^ and excelled at phagocytosis (Figure 7). However, we could perfectly, like the Powrie group (108), have classified these cells as moDCs based on their CD11c^hi^MHCII^hi^ profile, their localization within the T cell zone, their antigen-presentation capacity, or their dendritic morphology (Figure 7). By classifying these cells as CD64^hi^F4/80^hi^ and/or CD11c^hi^MHCII^hi^ MCs, they are recognized as one lineage, which will promote understanding and simplify communication between different research groups without preventing the study of DC-like or MΦ-like properties of specific MCs.

Finally, we do not expect MCs to be homogeneous in inflamed tissues. We and others found iNOS, the enzyme that is used by MΦs to produce NO and that is classically associated with an M1 activation state, to be typically expressed by only 10% of MCs (11, 45, 109). Since NO is bactericidal, suppressive for T cells, and can induce serious tissue damage, it makes perfect sense to study the factors that induce the expression of iNOS on a fraction of MCs. But by classifying these cells as “iNOS^+^ MCs” instead of “M1 macrophages” or “TIP-DCs” one avoids to associate functions that have not been proven for these cells such as the antigen-presentation activity typically expected from DCs [in fact mice lacking monocytes showed identical T cell priming suggesting that TIP-DCs are not essential for this function (11)].

## The Tough Cases Part I: moDCs as Fourth DC Subset?

MCs fitting the complete list of characteristics attributed to moDC, including migratory and antigen-presentation capacities comparable to cDCs, are not easily identified *in vivo*. We have described migratory MCs upon house-dust mite (HDM) exposure in the lungs (47), but their migration is much less efficient as compared to cDCs and required very high (and non-physiological) doses of HDM. In fact, we found that the majority of HDM-induced pulmonary MCs are not migratory cells but instead play an important role in the secretion of inflammatory chemokines that orchestrate the local immune responses. Similarly, a low-grade migration of CCR2^+^CD64^int^ MCs was described upon DSS inflammation in the skin but this was minor as compared to cDC migration (48). Moreover, compared to cDCs these dermal CCR2^+^CD64^int^ MCs displayed a rather modest antigen–antigen presentation capacity.

The most convincing pieces of evidence for cDC-like features of MCs come from *in vitro* culture systems. Bone-marrow cells cultured with GM-CSF yield cells with excellent antigen-presentation capacity that acquire CCR7, the chemokine receptor controlling migration of cDCs from tissues to lymph nodes, upon TLR stimulation and can migrate to the lymph nodes upon *in vivo* transfer (110, 111). This culture system has been used in many labs and is globally accepted to yield a homogeneous population of moDCs. This concept was first challenged by a study using single-cell transcriptomics (112). Among LPS-stimulated GM-CSF-induced bone-marrow-derived moDCs, the majority of cells were found to show high expression of inflammatory genes such as TNF, IL1, and CXCL10, while a smaller subset had much lower expression of these genes but displayed a signature reminiscent of “mature DCs,” including high expression of CCR7 (113, 114). This was originally interpreted as functional heterogeneity among moDCs. However, in what we consider a landmark paper, Reis e Sousa and colleagues now demonstrate that this minor “mature” population in fact represents cDC2s that contaminate these cultures. These cDC2s displayed lower production of inflammatory cytokines but much better CCR7-ligand-induced migration and antigen-presentation as compared to GM-CSF-induced MCs (115, 116). This implies that many of the DC-like features of GM-CSF-induced moDCs should in fact not be attributed to MCs, but to a minor contaminating cDC2 population. All in all, both *in vitro* and *in vivo* data thus point toward a lower migration and antigen-presentation capacity of MCs as compared to cDCs, but conversely a higher production of inflammatory cytokines and chemokines. We therefore propose that in an inflamed organ the core business of cDCs will be migration to the draining lymph nodes and activation of naïve T cells, whereas MCs will primarily orchestrate local inflammatory responses. Note that this has important consequences for DC-based vaccination strategies as this may explain why MC-based vaccines have only yielded modest clinical responses (117). The recent advances in cDC culture systems and the proper identification of committed circulating DC-precursors (31, 32, 118) may therefore pave the way toward more efficient cDC-based vaccination strategies.

## The Tough Cases Part II: Steady-State MCs Versus Embryonic Macrophages

Most MΦ-like cells present in steady-state tissues are of embryonic origin (33–36, 38, 39, 44). However, puzzling exceptions have been reported. Although embryonic MΦs colonize the intestine and the heart before birth, these cells are thereafter progressively replaced by MCs. Importantly, these cells are relatively short-lived and continuous monocyte-recruitment is required to maintain the MC pool in these tissues (45, 46, 119–121). Similarly, monocytes are continuously recruited to the steady-state dermis (48). Therefore, while in some steady-state tissues, including the lung and the spleen, monocytes have been proposed to remain in an undifferentiated state (37, 122); in others, including the intestine, the skin, and the heart, they acquire a MΦ-like phenotype. The classification of MCs that differentiate in these steady-state organs and that replace the embryonic MΦs is difficult. They do not fit the profile of the MCs that are recruited to inflamed tissues, including pulmonary infection (36), auto-immune brain inflammation (101, 102), and acute liver injury (100), since in these inflammatory settings MCs do not replace the embryonic MΦs and display a very different gene-expression profile. Future research will be required to compare the functional properties and gene-expression profile of the embryonic MΦs present in the intestine, the skin, and the heart to the ones from the MCs that replace them with time. It will be interesting to compare the influence of tissue-imprinting to the intrinsic differences associated with their distinct cellular origin. Embryonic MΦs were recently compared to their bone-marrow-derived counterparts that replace them after irradiation-induced depletion. It was found that both cells share between 50 and 90% of the tissue-specific epigenetic landscape (105). This emphasizes the importance of tissue-imprinting, but at the same time implies that between 10 and 50% of the epigenetic landscape could be governed by the cellular origin of the cells. Future research will be required to assess the functional relevance of these findings. In the meantime, the classification of MΦ-like MCs in steady-state tissues remains difficult.

## The Way Forward

The Level1 that forms the scaffold of the herein proposed classification system is in part based on elegant murine fate-mapping systems developed to study the cellular origin of MΦs (33, 34, 38, 39, 43, 44) and DCs [(123) and (124) in this issue]. Although the recent identification of committed DC-precursors distinct from monocytes in humans suggests that many of the principles identified in mice apply to the human immune system, this remains to be formally proven. Moreover, many of these murine fate-mapping systems label only a small fraction of the cells per population, rendering functional studies difficult. In cases where classification as cDC1, cDC2, pDC, MC, or MΦ is not obvious, a core set of signature genes that are specific for each cell type could facilitate correct Level1 identification. However, such signature genes are not easily identified. In addition, identification based on surface receptors would be most practical since it would allow the sorting of living cells through flow cytometry for functional assays. Ideally, such markers would be conserved across species. We are currently data-mining the gene-expression profiles of cells from various tissues and species to try to identify such markers. This can however represent a catch22. To find markers specifically expressed by the different populations, one requires pure gene-expression profiles, but correct sorting of the cells without contamination by other populations for RNA profiling requires the very markers we are looking for. Recent technological advances in single-cell RNA sequencing will allow to profile the gene expression of mixed populations. This may at last disentangle mixed myeloid populations and will hopefully provide the field with new markers that can then be validated with the current fate-mapping systems.

Although the current classification systems should thus be seen as work in progress, we are confident that in the near future better markers will be found which faithfully translate the cellular origin of cells and will form a practical base for the Level1 classification of myeloid cells. The Level2 classification should in our view be kept as flexible as possible to allow researchers to focus on one particular functional attribute of their cells of interest without implying too many additional features that have not been studied. Finally, it is noteworthy that in parallel to our proposition a nomenclature system for MΦs was proposed (106). In this proposition, terms implying functional specialization such as “classically activated macrophages” (pro-inflammatory) or “alternatively activated macrophages” (anti-inflammatory) were replaced by an objective description of how a MΦ is cultured *in vitro* [e.g., MΦ (IL-10)] or identified *in vivo* (e.g., “Relma^hi^ MΦ”). Thus, the common core Level1 would be MΦ and the added description provides the Level2. This and our classification system thus share three important principles: (i) elimination of terms that imply functional specialization as much as possible, (ii) introduction of a fixed Level1 system across species and tissues, and (iii) permitting flexibility through a Level2 system. Irrespective of which system is used to define the Level1 [ontogeny as we propose, or gene-expression profile as proposed by the Dalod group in this issue (60, 61)], we feel these three principles should be maintained for a future and hopefully definitive classification system.

## Conflict of Interest Statement

The authors declare that the research was conducted in the absence of any commercial or financial relationships that could be construed as a potential conflict of interest.
